# Evaluation of Learner Engagement Following Virtual Reality Augmented Central Line Training

**DOI:** 10.1089/jmedxr.2024.0058

**Published:** 2025-03-28

**Authors:** Margaret Sande, Kevin Livingstone, David Moody, David Martin, Kathryn Mutter

**Affiliations:** ^1^ Emergency Medicine, University of Virginia, Charlottesville, Virginia, USA.; ^2^ Emergency Medicine, Wake Forest University, Winston-Salem, North Carolina, USA.; ^3^ Health System Library, University of Virginia, Charlottesville, Virginia, USA.; ^4^ University of Virginia, Charlottesville, Virginia, USA.

**Keywords:** medical education, central line, procedural training, competency

## Abstract

Central line placement is a critical skill for medical students, yet training opportunities are limited. This pilot study evaluated the impact of virtual reality (VR) training on fourth-year medical students’ perceived competence for central line placement. Fifty-four students underwent VR training followed by hands-on simulation. Results demonstrated a significant increase in perceived competence post-training, with improvements across multiple dimensions. Students also rated the VR experience as highly valuable for their learning. While engagement with the VR interface was moderate overall, students found the technology appealing and worthwhile. These findings suggest that VR holds promise as a valuable adjunct to traditional central line training, enhancing students’ preparedness for clinical practice.

## Background 

Medical students face challenges learning essential procedural skills and are often not exposed to central line training during their education.^1^ Up to 42% of residents report a lack of training and are uncomfortable performing procedures such as a central line or other basic procedures alone for the first time.^2^ Fourth-year medical student training, which includes didactic instruction, simulation-based instruction, and observation at the patient’s bedside, can still feel insufficient in the development of skills and procedural confidence.

Virtual reality (VR) allows learners to expose and immerse themselves in a training environment without restrictions of time or place.^3^ In prior work, VR use in medical education has been shown among medical students to increase engagement, improve task performance tests, and may increase information retention.^4–7^ Thus, the possibility exists for VR to help fill the gap in central line training that currently exists in contemporary medical education. To that end, this project was a pilot innovation study to determine whether VR procedural training for central line placement improves students’ perceived competence in preparation to place a central line as our primary objective. A secondary objective was to determine whether students found the VR pre-learning engaging as well as valuable to their learning when combined with the hands-on training experience.

## Methods

### Ethics approval

Approval from the University of Virginia institutional review board for social and behavioral sciences was obtained prior to commencement of the study (IRB-SBS #5643).

### Enrollment

In March 2023, 60 fourth-year medical students enrolled in our medical school’s internship readiness course, and of those, 54 volunteered to participate in this study to evaluate a VR-enhanced central line training program as part of the course. Consent was obtained prior to enrollment. The central line program included a VR training experience 1–2 days prior to participating in a hands-on central line procedural training.

### VR training experience

The VR training environment was created *de novo* by the research team, with videotaping, programming, and ultimate compilation done by members of the research team. The VR training included a detailed step-by-step demonstration of central line placement on a mannequin with gamification enhancements via interspersed multiple-choice questions, pic-in-pic labeled live video of ultrasound landmark identification, step outline parallel display, and sequencing activities to support learning.^8^ Content is delivered via VR headset to allow for full immersion within the procedural suite while taking advantage of an expanded viewing environment and minimizing distraction. The expanded view allows supplemental visuals such as diagrams of relevant anatomy, live ultrasound pic-in pic, and interactive activities such as selecting from the kit the instrument for the subsequent step of the procedure (“choose the next piece of equipment needed” exercise) to not compete with the primary view of the proceduralist during central line placement. The learner’s vantage point is both “at the shoulder” and “birds eye” so as to provide the best view possible for instruction at each step. Likewise, the learner has full control over procedural progression with a table of contents to guide their experience and allow movement to review or progress as desired. Sample screenshots from the VR central line experience are included in Supplementary Appendix A. Of note, training was formative, and there was no grade or score associated with completion or denoting successful completion. Unlimited attempts ensured students answered questions correctly before progressing in the VR demonstration.

### Surveys

Prior to the VR training, baseline data were obtained on participant-perceived central line placement competence via an adapted Intrinsic Motivation Inventory (IMI), a validated study instrument. The students then completed the VR training experience. Immediately following completion of the VR training, students completed the User Engagement Scale short form (UES-SF) to assess their level of engagement with this technology. Approximately 1–2 days later, students were scheduled for a 90-min hands-on small group instructional session in which students practice central line placement on a task trainer with the guidance of a physician instructor. Immediately following completion of the hands-on instructional session, students were asked to complete two subscales of the IMI instrument—perceived competence and value/usefulness—as a means to compare before and after self-assessed competency. The specific questions asked for these two survey instruments are included in Supplementary Appendix B.

### Background of the survey instruments

The IMI is a multidimensional instrument developed and validated to assess participants’ perceived experience related to a specific activity.^9,10^ IMI has six subscales that can be used depending on the question being addressed: interest/enjoyment, perceived competence, effort/importance, pressure/tension, perceived choice, value/usefulness, and relatedness. Subscales have been shown to be stable across a variety of tasks, conditions, and settings. It is often modified slightly to fit specific activities. The perceived competence scale is theorized to be a positive predictor of both self-report and behavioral measures of intrinsic motivation. The value/usefulness subscale is used in internalization studies in evaluating how people internalize and become self-regulating with respect to activity that they experience as useful and valuable for themselves. This instrument was selected so that we could examine perceived competence pre/post completion of our multimodal training program.

The UES-SF is a validated instrument to measure engagement specifically with digital media such as virtual reality, video games, haptics, and other electronic applications and educational tools.^11^ It was originally released as a 31-question survey measuring 6 dimensions of engagement: aesthetic appeal, focused attention, novelty, perceived usability, felt involvement, and endurability, but follow-up work validated the shorter form utilized in our study. These survey tools were adapted with language oriented toward our activity and have been validated to be used in this manner.

The IMI for perceived competence was given prior to VR training and immediately after hands-on central line training. The UES-SF was administered immediately following completion of the VR experience. The IMI for value/usefulness was given following the hands-on central line training. Mean values were used for statistical analysis of the IMI for perceived competence, and mean values were obtained for the IMI for value/usefulness and UES-SF.

## Results

**FIG. 1.**
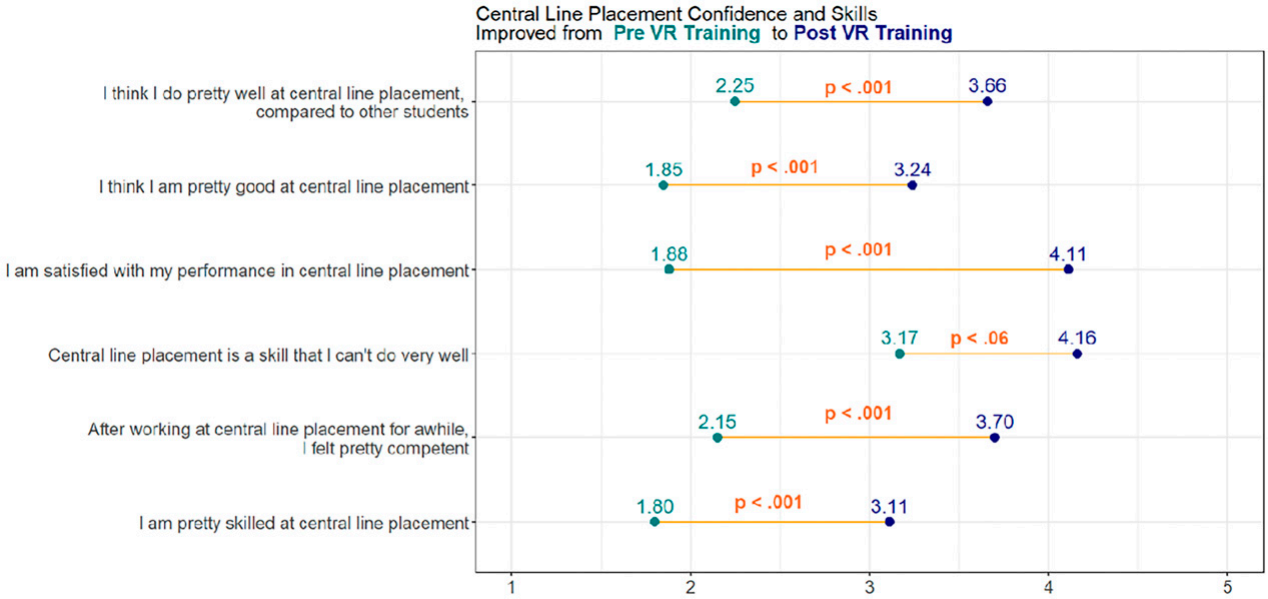
Pre versus Post Virtual Reality Training Perceived Competence, Intrinsic Motivation Inventory (IMI).

Of those enrolled, 54 learners completed the UES-SF, and 42 learners were assessed both collectively and as self-matched participants using the IMI, completing all instruments included in the study. Comparing the IMI perceived competence pre- and post-survey student responses indicated a statistically significant improvement in their perceived competence (*p* value <0.001). When assessing the six questions comprising the overall IMI for perceived competence, five out of the six showed significant improvement (Fig. 1). Likewise, when asked to assess the value of VR when coupled with hands-on instruction via the IMI for value/usefulness, responses were uniformly positive and ranged from 4.02 to 4.38 on a 7-point Likert scale with a mean of 4.22 (Table 1).

**Table 1.** Value/Usefulness Scores, Intrinsic Motivation Inventory (IMI)

VR device	*n*
Headset	42
Computer-based	5
I believe the immersive VR experience was of some value to me in being able to successfully learn central line placement	4.09
I think that doing the immersive VR experience is useful for learning central line placement	4.28
I think the immersive VR experience was important to do because it can help me learn central line placement	4.02
I would be willing to do this again because it has some value to me	4.38
I think doing the immersive VR experience could help me to remember key steps for central line placement	4.30
I believe doing the immersive VR experience was beneficial for me	4.36
I think the immersive VR experience is an important activity	4.13

VR, virtual reality

Interestingly, when asked about the user engagement with the VR interface via the UES-SF instrument, learners were more neutral about the technology itself as illustrated in Table 2 (range 2.69–3.81 on a 5-point Likert, mean 3.46). The answers that scored more closely to agreement around engagement were related to the technology being aesthetically appealing, attractive, worthwhile, and an interesting experience. Students did not find the technology taxing or confusing. Of note, 6 of the 54 learners (11%) reported nausea or discomfort of some type. Three out of 6 of these instances could have been corrected by adjusting the VR viewing configurations for the participant. Two learners stated in their open feedback questions that our “Zoom” level was too high and could have been corrected by the VR delivery team. A third learner commented about orientation issues such that they needed to look down for the duration of the experience. This too could have been corrected by the VR delivery team.

**Table 2.** Virtual Reality Interface User Engagement Scores via User Engagement Scale-Short Form (UES-SF)

VR device	*n*
Headset	54
Computer-based	1
I lost myself in this experience.	2.69
The time I spent using this VR experience just slipped away.	2.95
I was absorbed in this experience.	3.59
I felt frustrated while using the VR experience.	2.29
I found the VR experience confusing to use.	1.85
Using the VR experience was taxing.	2.26
The VR experience was attractive.	3.69
The VR experience was aesthetically appealing.	3.70
The VR experience appealed to my senses.	3.48
Using the VR experience was worthwhile.	3.69
My experience was rewarding.	3.59
I felt interested in this experience.	3.81

VR, virtual reality

## Discussion

Our data indicate that an interactive VR experience as prework to procedural training heightened learner engagement both in perceived competence and demonstrated value when paired with subsequent hands-on instruction. Open-ended feedback for the program as a whole supports this finding. Interestingly, students were more neutral about their engagement specifically with VR, though some answers trended toward agreement surrounding finding the VR training attractive, aesthetically appealing, worthwhile, and an interesting experience. This may be explained by several factors. Individual learning styles differ, and VR capitalizes on game-based learning and self-directed learning, whereas other students may find greater value in hands-on simulation instruction with kinesthetic and experiential learning pedagogies. In support of this point, some participants reported a desire for the VR experience to allow the end user to perform the central line themselves, unfortunately not feasible with the technology constraints of the project and budget. While it is possible that the competency benefit was primarily driven by the hands-on simulation session, it is also plausible that the VR experience may have had more impact than students realized as a preparatory tool leading to higher yield use of time during the hands-on session. Finally, several study participants reported difficulty with the VR secondary to motion sickness while wearing the headset.

There are limitations to this study. We were unable to isolate the impact of VR versus hands-on simulation instruction to evaluate the relative impact of each component given logistical challenges of the clinical rotation. However, we did ask students to report their perceived value of the VR in preparing for the central line placement session, and they noted that there was value, as it was felt to be beneficial for preparation and helped them remember the steps of the procedure. A second limitation of this study is that we examine perceived competence rather than externally observed and measured competence. Finally, we were unable to account for learner-preferred pedagogies either collectively or at the individual level nor predetermine those with a history of cybersickness to personalize their learning experience.

Future work should include evaluating VR in isolation, both with and without various gamification elements, to explore the relative impacts of each. Additionally, VR could also be compared to relative impact on preparation for hands-on instruction when compared to comparable 2D video. Finally, future investigations could incorporate using validated competency instruments in central line placement to examine the impact of training with and without VR as a precursor.

## Conclusions

In conclusion, this study demonstrated that a VR training program, when paired with hands-on central line training, showed a statistically significant improvement in students’ perceived competence in placing central lines and was deemed valuable in anchoring their training. Further studies are required to delineate specifically what is driving these increases and whether externally validated competence correlates. Given our positive findings, we plan to continue to create a library of VR procedural training experiences that could be accessed by learners interested in finding new modalities to improve their procedural preparation.

## Abbreviation used

VR: virtual reality
IMI: Intrinsic Motivation Inventory
UES-SF: User Engagement Scale short form
