# Supplementary material for: Evaluation of Learner Engagement Following Virtual Reality Augmented Central Line Training
Source: J Med Ext Real. 2025 Mar 28;2(1):76–80. doi: 10.1089/jmedxr.2024.0058 (PMC13387241; doi:10.1089/jmedxr.2024.0058)
Supplement: Supplementary file 1 [file jmedxr.2024.0058_supplementary_appendix_a.docx]

Appendix A. Representative Images of VR Augmented Central Line Training Experience

A. ‘Choose the next piece of equipment needed’ Exercise

B. Gamification via real-time question integration. Guided feedback provided for erroneous answers; correct answer required to advance.

C. Learner participating in VR central line experience; Live ultrasound pic-in-pic with an associated multiple choice question.
